# A Novel Capacity-Strengthening Intervention for Frontline Harm Reduction Workers to Support Pre-exposure Prophylaxis Awareness-Building and Promotion Among People Who Use Drugs: Formative Research and Intervention Development

**DOI:** 10.2196/42418

**Published:** 2023-04-13

**Authors:** Jennifer L Glick, Leanne Zhang, Joseph G Rosen, Karla Yaroshevich, Bakari Atiba, Danielle Pelaez, Ju Nyeong Park

**Affiliations:** 1 Department of Health, Behavior, and Society Bloomberg School of Public Health Johns Hopkins University Baltimore, MD United States; 2 Department of International Health Bloomberg School of Public Health Johns Hopkins University Baltimore, MD United States; 3 Charm City Care Connection Baltimore, MD United States; 4 Division of General Internal Medicine Warren Alpert Medical School Brown University Providence, RI United States; 5 Center of Biomedical Research Excellence on Opioids and Overdose Rhode Island Hospital Providence, RI United States

**Keywords:** formative research, harm reduction, intervention development, pre-exposure prophylaxis (PrEP), people who use drugs (PWUD)

## Abstract

**Background:**

HIV prevalence among people who use drugs (PWUD) in Baltimore, Maryland, is higher than among the general population. Pre-exposure prophylaxis (PrEP) is a widely available medication that prevents HIV transmission, yet its usefulness is low among PWUD in Baltimore City and the United States. Community-level interventions to promote PrEP uptake and adherence among PWUD are limited.

**Objective:**

We describe the development of a capacity-strengthening intervention designed for frontline harm reduction workers (FHRWs) to support PrEP awareness-building and promotion among PWUD.

**Methods:**

Our study was implemented in 2 phases in Baltimore City, Maryland. The formative phase focused on a qualitative exploration of the PrEP implementation environment, as well as facilitators and barriers to PrEP willingness and uptake, among cisgender women who use drugs. This work, as well as the existing literature, theory, and feedback from our community partners, informed the intervention development phase, which used an academic-community partnership model. The intervention involved a 1-time, 2-hour training with FHRWs aimed at increasing general PrEP knowledge and developing self-efficacy promoting PrEP in practice (eg, facilitating PrEP dialogues with clients, supporting client advancement along a model of PrEP readiness, and referring clients to PrEP services). In a separate paper, we describe the conduct and results of a mixed methods evaluation to assess changes in PrEP-related knowledge, attitudes, self-efficacy, and promotion practices among FHRWs participating in the training.

**Results:**

The pilot was developed from October to December 2021 and implemented from December 2021 through April 2022. We leveraged existing relationships with community-based harm reduction organizations to recruit FHRWs into the intervention. A total of 39 FHRWs from 4 community-based organizations participated in the training across 4 sessions (1 in-person, 2 online synchronous, and 1 online asynchronous). FHRW training attendees represented a diverse range of work cadres, including peer workers, case managers, and organizational administrators.

**Conclusions:**

This intervention could prevent the HIV burden among PWUD by leveraging the relationships that FHRWs have with PWUD and by supporting advancement along the PrEP continuum. Given suboptimal PrEP uptake among PWUD and the limited number of interventions designed to address this gap, our intervention offers an innovative approach to a burgeoning public health problem. If effective, our intervention has the potential to be further developed and scaled up to increase PrEP awareness and uptake among PWUD worldwide.

## Introduction

HIV prevalence among people who use drugs (PWUD) remains high, including both people who inject drugs (PWID) and those who use drugs by other routes of administration [1-3]. Despite overall decreasing trends in new HIV diagnoses among PWID in the United States, estimates posit a 6%-10% HIV positivity among PWID [1,4] and there have been notable recent HIV outbreaks among this population [5]. Lowering the annual number of new HIV infections among PWID is a critical component of the US strategy to end the HIV epidemic [1].

There are high numbers of PWID in Baltimore, many of whom engage in polysubstance use [6-8]. Baltimore had the sixth highest PWID HIV prevalence among participating cities in Centers for Disease Control and Prevention’s (CDC) 2018 National Behavioral Surveillance System, with a 10% HIV positivity among the full sample, higher rates among men (11%) than women (7%), and the highest HIV positivity (17%) among African Americans nationally [9]. PWID reported high rates of receptive sharing of syringes (33%) and equipment (59%) [9].

HIV infection occurs when viral particles enter the bloodstream, most commonly via sexual or injection-mediated exposure, and infect the cells of the immune system. If left untreated, HIV infection may progress to AIDS; people with HIV who receive effective treatment can live long healthy lives and prevent onward transmission. Although there is not yet a vaccine to prevent HIV infection, medication exists to prevent successful infection of the immune system even in the event of exposure to the HIV virus. This medication, pre-exposure prophylaxis (PrEP), is a user-controlled pharmaceutical method that provides an empowering and highly effective means of preventing HIV in vulnerablized populations when taken as prescribed. PrEP reduces the risk of HIV acquisition via sexual transmission by approximately 99% and from injection drug use by at least 74% [10]. However, adherence is crucial for successful risk reduction [11]. Postexposure prophylaxis is the use of antiretroviral drugs after a single high-risk event to stop HIV seroconversion [10].

In 2014, the CDC released clinical guidance recommending the use of PrEP for persons at increased risk of acquiring HIV [12]. However, PrEP awareness and uptake among PWUD remain low. For example, a global systematic review of women who use drugs reported low awareness of and willingness to use PrEP, ranging from 42% to 89% [13]. Findings among PWID of all genders show low levels of accurate PrEP knowledge and discordance between reported risk behavior and risk perception, which informs PrEP interest [14-16]. The results are conflicting regarding the role of gender and gender disparities in PrEP uptake among PWUD [13]; some studies found no association between gender and acceptability [17,18], whereas others found that women were more willing to use PrEP than men [19,20]. In Baltimore, only one-fourth (27%) of HIV-negative PWID were aware of PrEP, and only 1% of HIV-negative PWID reported taking PrEP; awareness rates were comparable among men and women [9,21].

Interventions to address PrEP awareness, uptake, and adherence among PWUD are limited [22]. Preliminary data are emerging concerning factors that impact PrEP awareness and uptake among this population [14,15,22]. Broadly speaking, PWUD face barriers accessing health services, reporting negative stigmatizing experiences seeking medical care, commonly seeking care in emergency departments, and may be less likely to encounter a provider willing to discuss PrEP [20,23-25]. PrEP intervention strategies centering non–health care settings show preliminary success, demonstrating that proximity to harm reduction services and colocation of services are important facilitators. For example, women who inject drugs (WWID) who reported syringe exchange program usage were more likely to be PrEP aware and to initiate and adhere to PrEP [26,27]. Although these results are promising, more work is needed to inform critical gaps in HIV prevention and PrEP promotion among PWUD.

To address this gap, the Optimizing PrEP Engagement Among Women Living in Baltimore City (OPAL) study was initiated with the goal of developing a PrEP intervention for WWID by (1) conducting formative research with service providers and WWID to guide intervention development and (2) developing, piloting, and evaluating an intervention that addressed emergent barriers to PrEP uptake in this population. The study was expanded to encompass PWUD of all gender identities.

## Methods

### Study Design

**Figure 1:**
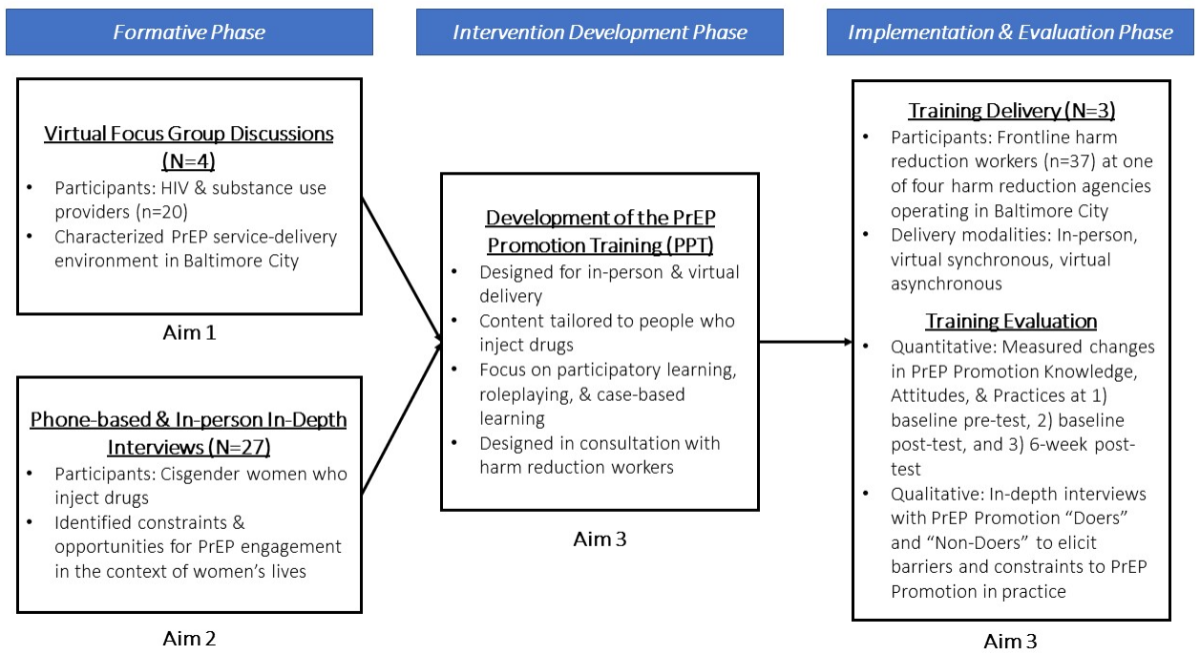
Study design diagram. PrEP: pre-exposure prophylaxis.

The OPAL study was a multiphase, sequential mixed methods formative research study (Figure 1) conducted in Baltimore City that was aimed at improving PrEP uptake among WWID and PWUD [28,29].

Understanding the complex lived experiences and challenges faced by WWID to inform intervention development requires a combination of methods drawing from different perspectives and epistemologies. A hybrid inductive-deductive qualitative approach is ideally suited for such an endeavor. In the formative phase, we facilitated 4 web-based focus group discussions with 20 HIV and substance use service providers who served PWUD to characterize the PrEP implementation environment in Baltimore City [30]. The OPAL study design integrated insights gleaned independently across study aims through data integration [31]; the findings from this first aim guided our sampling plan and research questions for the study’s second aim, in which we conducted semistructured in-depth interviews and brief surveys with WWID, exploring facilitators and barriers to PrEP willingness and uptake in the context of women’s substance use, health care engagement, and access to harm reduction services. Taken together, this formative work guided the intervention development phase, which was further informed by the existing literature and input from our community partners.

### Study Setting

All study activities were conducted in Baltimore, Maryland, which is a midsize urban center on the Eastern coast of the United States.

### Conceptual and Theoretical Foundation

The intervention development approach centered a combination of community participatory [32,33] and human-centered design [34] philosophies. Both approaches prioritize meaningful participation in the entire development process from those that will ultimately be the recipients of an intervention. These approaches prioritize consideration of “end-user” wants and needs in the process, as well as ensuring that participation is equitable and respectful. Our process included community partners in many aspects of the project, including formative research design and implementation; intervention conceptualization, design, and evaluation; as well as results interpretation and dissemination.

**Figure 2:**
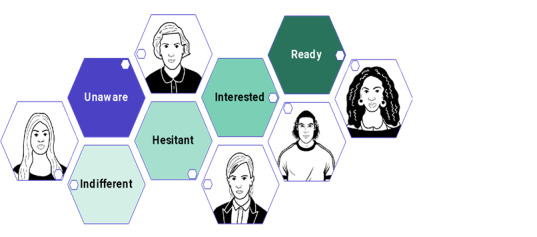
The pre-exposure prophylaxis awareness mosaic.

Furthermore, intervention component development was theory driven. For example, the role-play activities included in the trainings were informed by the social learning theory, which posits that human behavior is learned observationally [35], and the mosaic model (Figure 2) was built on an adaptation of the transtheoretical model of behavior change, which posits that behavior change happens via a progression through a series of stages [36].

Finally, the process and team members were motivated by harm reduction philosophies, which promote nonjudgmental orientations toward stigmatized behaviors, respecting personal autonomy, practicality, and innovation [37]. For example, the intervention centered PrEP “promotion” that we operationalized with a focus on expanding awareness and supporting uptake, only as desired by PWUD. We did not endeavor to facilitate PrEP uptake among all eligible PWUD, acknowledging that PrEP may not be an appropriate HIV prevention intervention for a given individual for a myriad of reasons, or may not be a current priority for PWUD.

### Conceptual Framing of Target Population

This project was initially conceived of as a way to investigate and meet the needs of WWID, given their unique risk factors [38,39] and exacerbated substance use outcomes [40,41]. Women are an underexamined group of drug users; frequently, research with PWUD is not disaggregated by gender [42,43]. However, given that much of the harm reduction work in Baltimore City is not explicitly implemented through a gender lens or targeted at gender-specific populations, we developed our intervention to center the education and skills development of frontline harm reduction workers (FHRWs) to support clients of all genders. To ensure our intervention centered the needs of women, we included educational information about the unique PrEP needs of cisgender and transgender women.

### Intervention Development Team

The intervention development team used an academic-community partnership model. The academic research team comprised faculty and student researchers from Johns Hopkins Bloomberg School of Public Health. The research team had experience with relevant research methodologies (formative research, intervention development, community-engaged qualitative and quantitative methods), the study topic, and the population of focus. Student members of the team received skills development training to make meaningful contributions in their respective roles. The team ranged in gender, race, sexual orientation, age, and educational preparation.

The community partners included staff from Charm City Care Connection, a community-based harm reduction organization in Baltimore City that works to connect those impacted by drug use to high-quality health care services and address any obstacles that might threaten that connection. Their mission is to promote health, self-determination, and self-advocacy for individuals and communities affected by drug use, stigma, poverty, and inequities, with a primary focus on serving PWUD. Provided services include, but are not limited to, safer use supplies (eg, syringe services, sterile smoking kits), overdose education and prevention materials (eg, naloxone, fentanyl test strips), safer sex supplies, peer recovery services, case management, miscellaneous services for basic life necessities (ie, food and clothing), a drop-in center for PWUD, street-based outreach, and a small housing program. Charm City Care Connection also operates a Harm Reduction and Anti-Oppression Training Program, where PWUD can volunteer at the drop-in center and participate in workshops that focus on personal success planning, anger management, conflict resolution, trauma and crisis management, healthy relationship building, and self-advocacy. Once these trainings are completed, trainees are offered the opportunity to work as part-time employees, providing the same services that they once received to their peers.

### Formative Research and Collaborative Intervention Development Process

The activities of the formative research phase were designed, developed, and implemented by the academic research team. Logistical support during this phase was provided by the community partner and included linking clients with study staff for interviews. Upon completion of the formative phase, findings were shared with the partner organization, who emphasized that FHRWs felt underequipped having conversations about PrEP with PWUD due to a lack of knowledge and low self-efficacy associated with the sensitive nature of HIV-related discussions. Ongoing conversation regarding formative findings, interpretation, and implications within the academic research team led to the conceptualized idea to develop a PrEP promotion training (PPT) for FHRWs. This concept was presented to representatives of the partner organization, who endorsed the concept and agreed to participate in the development and implementation process.

**Figure 3:**
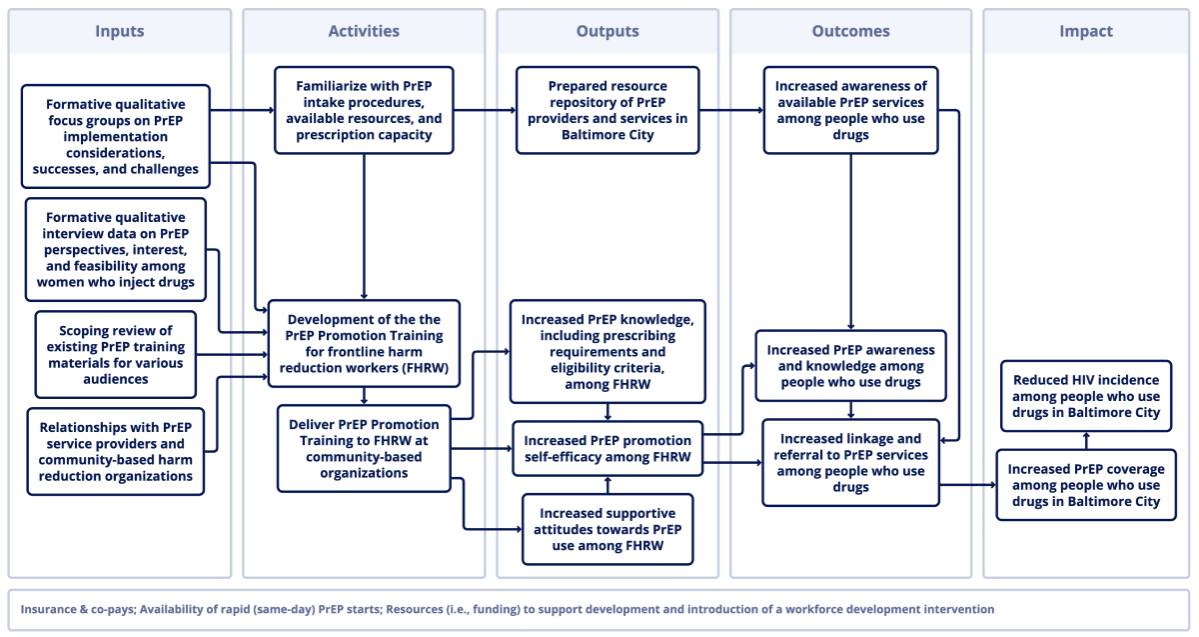
Intervention logic model. FHRW: frontline harm reduction workers; PrEP: pre-exposure prophylaxis; PWUD: people who use drugs; WWID: women who inject drugs.

To guide the intervention development process, the academic research team developed a logic model for the intervention, which included the formative research and project work (inputs), ultimately with an aim of reducing HIV incidence among PWUD (impact) by increasing PrEP awareness and relevant skills among FHRWs (outputs), which would subsequently increase PrEP awareness, knowledge, and linkage to services among PWUD (outcomes) (Figure 3).

The training content was developed by members of the academic research team in consultation with the partner organization representatives, who provided input and feedback to ensure that content was relevant to their work, representative of their experiences with clients, and digestible by prospective training participants. For example, feedback from the partner organization spurred the academic research team to include role-play exercises and other opportunities for active learning and engagement in the training design.

### Formative Research Findings Related to Intervention Conceptualization and Development

Findings from focus group discussions [30] underscored the importance of colocating PrEP delivery with other health and social services accessed by PWUD, including mobile harm reduction (ie, syringe services), medication for opioid use disorder, and mental health care. Providers also emphasized the role of service provider continuity for client rapport-building and relationship maintenance to support PrEP interest and uptake.

Interviewed WWID exhibited moderate PrEP awareness overall, with most having been introduced to PrEP through television commercials or advertisements. Few women reported ever discussing PrEP with a health care provider. Many WWID reported that HIV risk management did not feature as prominently in their everyday lives as other health and survival priorities, such as managing withdrawal symptoms or earning money. However, many women described engaging in HIV risk reduction through condom and sterile syringe use, which they received from FHRWs. Furthermore, many WWID reported routinely accessing harm reduction services in outreach settings (mobile- or street-based) or from drop-in centers.

Taken together, these findings highlight the existing proximity of FHRWs to WWID in Baltimore City and their potential to facilitate PrEP promotion among WWID. FHRWs already support these women and are well situated to promote reflection on HIV risk, initiate conversations about PrEP, and support clients in accessing PrEP if and when ready.

### Intervention Participants: FHRWs

The intervention was tailored to the experiences, knowledge, and client interactions typical of FHRWs at community-based organizations in Baltimore City that offer both outreach and drop-in services and do not have standing PrEP programs onsite. FHRWs were chosen as the study population due to their proximity to and strong relationships with PWUD, developed through existing motivational interviewing skills and regular client interactions. Furthermore, many FHRWs have lived experience with drug use and other adversities faced by PWUD, which facilitates connection and helps to overcome issues establishing trust and rapport frequently reported by PWUD when accessing services in traditional health care settings [20,23]. This makes FHRWs uniquely positioned to inform and link PWUD to PrEP services from outside the health care setting. Further, FHRWs have consistent opportunities to talk about infection prevention with clients, through syringe service provision, condom distribution, and STI and HIV testing services. Additionally, established trust between clients and FHRW facilitates ease in potentially sensitive or stigmatizing conversations about sex and injection practices.

### Intervention Description

The PPT was delivered as a 2-hour training divided into 2 sections: the first section was didactic, presenting basic information about PrEP (eg, the medication’s purpose, relevance for client populations, effectiveness, side effects) and steps for making referrals to local PrEP services (Textbox 1). PrEP information was presented in a demedicalized fashion and was specifically tailored to be relevant to populations served by participating organizations (eg, PrEP compatibility with gender-affirming hormones, Hepatitis C treatment). We used nonclinical language to share PrEP information, ensuring that the content was accessible to FHRWs with varying degrees of technical expertise. The goal of the didactic component was to increase FHRWs familiarity with PrEP such that they could initiate PrEP discussions with clients, answer basic questions about PrEP, and refer clients to PrEP services and resources in Baltimore City.

**Intervention modules.**

**Pre-exposure prophylaxis (PrEP) 101 (*didactic*)**

- Overview of HIV risk, transmission dynamics, and prevention
- PrEP mechanisms of action, coverage indicators, and efficacy
- Current PrEP formulations and modalities with Food and Drug Administration approval
- Populations that could benefit from PrEP
- Clinical and use requirements for PrEP effectiveness
- PrEP safety and side effects
- PrEP versus postexposure prophylaxis
- Available PrEP services in Baltimore and information resources

**Raising awareness and promoting PrEP (*practice*)**

- The continuum of PrEP awareness and readiness (mosaic)
- Introducing and reviewing client personas
- Strategies for introducing PrEP into client dialogues
- Role play exercises: (1) raising awareness, (2) answering questions, and (3) supporting PrEP uptake
- Supporting continuity in PrEP dialogue with clients
- Questions and feedback

The second portion of the training involved several active learning activities designed to enhance FHRWs self-efficacy promoting PrEP in practice. First, we introduced the concept of a “PrEP awareness mosaic” (Figure 2), an internally developed model of PrEP awareness that situated PrEP interest into a nonlinear continuum, with differently colored “tiles” representing different states of awareness, interest, or readiness in PrEP (“Unaware,” “Indifferent,” “Hesitant,” “Interested,” and “Ready”). The mosaic was designed to underscore that participants may encounter clients at various “tiles” of the mosaic and should adjust their PrEP messaging accordingly, and PrEP conversations should aim to increase PrEP awareness and support clients’ PrEP-related wants and needs based on whichever mosaic tile they may fall on, rather than facilitate directional momentum along a PrEP continuum that may not be aligned with the clients’ wants and needs.

We developed 5 client “personas” (or archetypes), loosely informed by the composite lived experiences and PrEP willingness of interviewed WWID in the study’s formative research phase (Figure 1) [44]. These personas were reviewed by community partners to ensure that they realistically represent the types of clients that FHRWs encounter. We presented 3 personas in each training as a pedagogical tool to help FHRWs practice identifying a theoretical client’s pre-existing “mosaic tile” level of PrEP awareness and anticipated enabling or constraining factors to PrEP willingness and uptake. During the training, participants read a brief (approximately 5 sentences) description of each persona’s lived experiences (patterns accessing harm reduction services, HIV risk, substance use, health care engagement, experiences with PrEP) and collectively discussed where on the PrEP awareness mosaic the client may fall, approaches to increase or enhance PrEP knowledge, and tools for supporting anticipated PrEP needs (eg, questions, referrals) among clients.

After discussing client personas, we discussed strategies for introducing PrEP into dialogues with clients encountered during drop-in services or during mobile outreach. This included identifying both simple entry points (ie, HIV testing encounters, request for condoms and lubricant) and more ambitious targets (ie, syringe and overdose prevention services, drug checking) for introducing PrEP during client interactions.

We then facilitated a roleplay activity in which training participants were paired up and practiced skills for 3 different types of PrEP conversations: identifying clients’ knowledge of PrEP, answering clients’ questions about PrEP, and linking clients to PrEP services. During each roleplay exercise, participants played the role of either a FHRW or 1 of the 5 client personas. After each roleplay exercise, select pairs volunteered to present their roleplay dialogues to the other training attendees, allowing all participants to observe, reflect upon, and troubleshoot strategies for PrEP-related conversations.

At the end of the training, we facilitated a short debrief session to elicit feedback on the content and delivery of the PPT, identifying favorable attributes of the training as well as opportunities to modify training content and delivery.

In addition to sharing training resources (slide deck, a shortlist of PrEP frequently asked questions and responses, PrEP information resources), we provided PrEP information flyers containing basic information about PrEP and local venues where PrEP information and services could be accessed, adapted from the Maryland Department of Health’s PrEP toolkit [45]. We instructed training participants to share these flyers with clients, using them for initiating PrEP conversations, as appropriate.

### Evaluation

Given the novelty of the intervention, we conducted an evaluation to assess changes in PrEP-related knowledge, attitudes, self-efficacy, and promotion practices among FHRW participating in the training [29]. Using 3 structured surveys, we collected quantitative data before the training (baseline pretest), immediately following the training (baseline posttest), and 6 weeks after the training (endline posttest) to quantify the intervention’s effectiveness on key outcomes. Using self-reported PrEP promotion practice data at endline posttest, we segmented FHRWs into 2 groups: “doers” (those who self-report discussing PrEP with any client in the past month) and “nondoers” (those who self-report not discussing PrEP with any client in the past month) and sampled each group for participation in semistructured in-depth interviews, assessing barriers and facilitators to PrEP promotion. Participants who completed the evaluation received financial compensation (up to US $65) for their time.

### Ethical Considerations

The Johns Hopkins Bloomberg School of Public Health Institutional Review Board reviewed and deemed the study protocol as nonhuman subject research. The study team took all appropriate cautions to maintain data confidentiality by redacting all personally identifiable information from in-depth interview transcripts and generating unique study identifiers, which were linked to participant information only through a secure, password-protected file.

## Results

The intervention development occurred from October to December 2021. Intervention piloting began in December 2021 and was completed in January 2022.

### Intervention Participant Recruitment

The academic research team leveraged existing relationships with community-based harm reduction organizations to recruit FHRWs to attend the training and participate in the evaluation. We engaged 4 nonprofit organizations in Baltimore City offering low-threshold harm reduction services to various populations. The populations served by these organizations’ missions included transgender individuals, PWUD, people with unmet mental health needs, and women who sell sex. Community partners all offered drop-in as well as outreach (ie, mobile) harm reduction services, which included syringe and overdose prevention services, HIV and Hepatitis C testing, linkage to health care and safety net programs, among others. Shared technical expertise among staff at organizations included case management, service navigation, motivational interviewing, and harm reduction promotion.

### Intervention Sample

A total of 39 FHRWs from 4 community-based organizations participated in the training across 4 implementations (1 in person, 2 online synchronous, and 1 online asynchronous). FHRW training attendees represented a diverse range of work cadres, including peer workers, case managers, and organizational administrators. Attendees ranged in terms of racial and gender identities. Prior to the training, only two-thirds of participants were familiar with PrEP.

### Changes to the Intervention Setting

We initially designed the PPT to be delivered in person. In December 2021, we successfully facilitated our first in-person training with a community organization. However, due to surges in COVID-19 cases in January 2022, we pivoted to online delivery of the remaining trainings via Zoom. This transition required retooling of the practice-based training portion. To approximate the active participation and partner-based work involved with the in-person training, we used an integrated polling feature, conducted short knowledge checks, and encouraged the use of response and feedback tools (ie, thumbs up and down reactions, hand-raising function) to facilitate participant engagement throughout the web-based sessions. Web-based delivery also enabled us to deliver the training to more than 1 community organization in the same session, facilitating cross-organizational learning. A consequence of the shift to web-based delivery was hampered accessibility; less technically proficient FHRWs had difficulty in joining the videoconference session and using the web-based tools. To address this, we created a recording of 1 training delivery session and made it available to FHRWs who were not able to participate in live sessions.

### Evaluation

Evaluation activities began at the onset of the intervention pilot (December 2021) and were completed in March 2022. Of the 39 FHRW participants, 37 met the eligibility criteria and were included in the evaluation. The evaluation results were published in a companion article [29].

## Discussion

This intervention offers a novel approach to HIV prevention among PWUD, supporting FHRWs in initiating PrEP conversations and supporting client advancement along a PrEP continuum, as appropriate. The subsequent evaluation provides information on intervention effectiveness—preliminary feedback is promising [29]. Given the current lack of PrEP uptake among PWUD at risk of HIV, and the limited number of interventions designed to address this gap, our intervention offers an innovative approach to a growing public health problem.

This intervention takes a community-situated approach to HIV prevention among PWUD by focusing on and expanding the robust skillsets of FHRWs, which is a cadre of the health care and harm reduction workforce that has been underutilized in PrEP promotion approaches. FHRWs are particularly well situated and skilled to support PWUD in adopting additional harm reduction approaches, namely increasing PrEP-related general knowledge and services, given their role as trusted and accessible allies [20,23]. In fact, studies have shown that harm reduction service engagement is associated with higher PrEP awareness and uptake among PWUD [20,26,27]. Furthermore, although published interventions colocating PrEP and harm reduction services are minimal, a few existing interventions with WWID demonstrate that this integration shows promising results [27,46,47]. For example, a New York City–based pilot intervention that included the provision of brief PrEP education and PrEP care navigations at mobile and drop-in harm-reduction sites from peer outreach workers showed positive results in PrEP interest and appointment setting, but minimal appointment attendance and no PrEP prescriptions, highlighting a gap between PrEP interest and connecting women to PrEP care [47]. Another intervention integrating PrEP into drop-in services at a major syringe services program in Philadelphia reported high PrEP uptake (~66%) among women who were offered PrEP onsite [27]. Although the above studies focus on WWID, our study shows that the same may be true for PWUD of all genders.

The proposed intervention is not without potential obstacles and limitations. For example, although capacity building and skills development are integral to the ongoing success of harm reduction organizations, the addition of new services may be burdensome to existing operations. Harm reduction organizations are already working tirelessly, frequently with insufficient resources, to address overdose prevention and syringe exchange needs of PWUD. Although HIV and other infectious disease prevention is an implicit goal of this work, the addition of PrEP promotion can add additional operational considerations to an already-taxed workforce. However, harm reduction organizations are already adept at remaining nimble to policy changes and environmental shifts to ensure that they are meeting their mission. This is evidenced by the pivoting and rapid service adaptations exemplified by harm reduction organizations during the COVID-19 pandemic [48,49]. Our vision is that policy makers will see the value in expanding the breadth and scope of the harm reduction workforce and provide additional resources for such organizations to grow and expand in line with their tremendous potential for creating change among vulnerabilized populations.

### Conclusions

This intervention has the potential to be further developed and expanded to increase PrEP awareness and uptake among PWUD in a range of settings. After the achievement of conclusive results, we plan to refine and scale up the intervention. Our long-term vision is to develop this intervention with modifiable content units that can be tailored to specific subgroups of PWUD as needed, such as PWUD who exchange sex, of different gender identities, from different regions or political contexts, and residing in jurisdictions with variable PrEP service delivery infrastructure. Furthermore, such an intervention could be adapted to address additional HIV prevention and care services, such as HIV counseling and testing, HIV care and treatment, and emerging new technologies such as long-acting injectable PrEP.

## Abbreviations

CDC: Centers for Disease Control and Prevention
FHRW: frontline harm reduction worker
OPAL: Optimizing Pre-exposure Prophylaxis Engagement Among Women Living in Baltimore City
PPT: pre-exposure prophylaxis promotion training
PrEP: pre-exposure prophylaxis
PWID: people who inject drugs
PWUD: people who use drugs
WWID: women who inject drugs

## Data Availability

All data generated or analyzed during this study are included in this published article.
